# Pancreatic CSCs and microenvironment

**DOI:** 10.18632/genesandcancer.80

**Published:** 2015-09

**Authors:** Matthias Ilmer, David Horst

**Affiliations:** Department of General, Visceral, Transplantation, Vascular and Thoracic Surgery, Hospital of the University of Munich, Ludwig-Maximilians-University (LMU) Munich (Germany)

**Keywords:** CSCs, WNT, R-Spondin, pancreatic ductal adenocarcinoma, EMT

Pancreatic ductal adenocarcinoma (PDAC) is the most common malignant neoplasm of the pancreas and presents with dismal prognosis. Key hallmarks of these tumors are chemo resistance and early metastasis, leaving few effective options for curative treatment. In part, treatment resistance might be spawned by tumor cell plasticity within pancreatic cancers, and specifically by therapy resistant pancreatic cancer stem cells. The concept of cancer stem cells (CSCs) postulates that tumors are heterogeneous and hierarchically organized, with CSCs at their apex, aggregating conspicuous abilities such as self-renewal, recapitulation of the primary tumor and its propagation, pronounced generalized therapy resistance, and metastasis formation. Thus, understanding the nascency and regulation of pancreatic CSCs might be critical to find more effective treatment options for PDAC.

**Figure 1:**
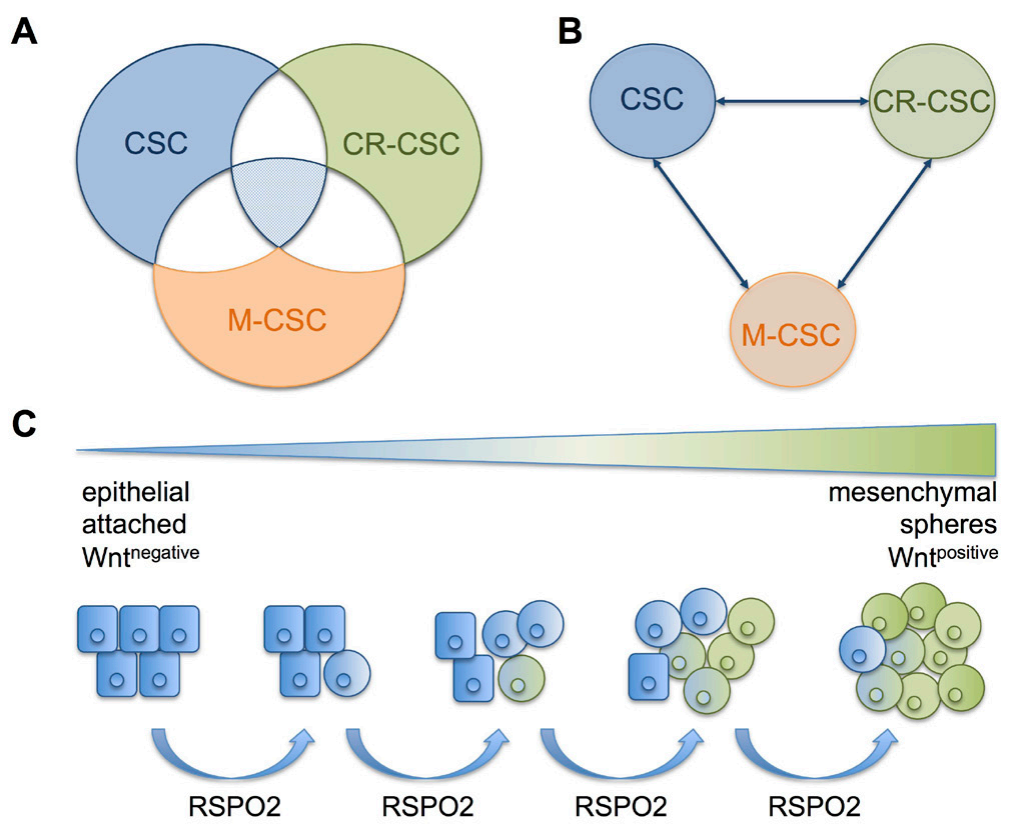
Relation of different CSCs and the influence of contextual cues of the CSC niche (A) Predefined states of different CSC types that partially overlap in functional abilities. Cancer stem cells (CSCs, *blue*), chemo resistant CSCs (CR-CSCs, *green*), and metastasizing CSCs (M-CSCs, *orange*). Overlap of two abilities are indicated in white ellipses, all three abilities are shown in the triangular intersection (*light blue*). (B) In contrast, fluctuating and interchangeable states of CSCs can be seen. CSCs can easily turn into CR-CSCs or M-CSCs and vice versa, depending on the contextual signals provided by the respective niche. (C) In the model system published recently [6], we found RSPO2 to be a trigger driving CSCs in susceptible pancreatic cancer cells, turning phenotypically epithelial and attached cells into phenotypically mesenchymal, detached and floating spheres.

The operational definition of CSCs relies on their ability to initiate tumor growth in xenograft models and thus recapitulate original tumor architecture. The cell surface marker CD133, and more efficiently the combination of CD133 and CXCR4, seemed to enrich for such CSCs that also showed high metastatic potential [1]. Moreover, functional properties, such as aldehyde dehydrogenase (ALDH) activity, anchorage-independent growth in serum-depleted media or, more recently, intracellular autofluorescence [2], have proven to effectively enrich for tumor initiating CSCs. However, these biomarkers remain unreliable and, despite many research efforts, CSC regulation still is incompletely understood. In this context, current research focuses on the question if cancer stemness is a rigid predefined or a fluctuating state with susceptible “stemness-prone” cells that are able to switch between CSCs and non-CSCs due to tumor cell plasticity. Additionally, certain subtypes of CSCs might be more relevant for metastasis (M-CSCs) or confer chemo resistance (CR-CSCs), while both properties may overlap (Figure 1A and B) [3].

CSC properties may depend on certain signaling pathway activities and in this context, WNT signaling has been attributed a role in colorectal cancer [4]. Although canonical WNT signaling is generally not affected by driver mutations in PDAC, recent evidence suggests that it might be required for early tumorigenesis in PDAC [5]. Most likely, this (temporary) activation of WNT is ligand-receptor mediated and limited to physiological levels of WNT in contrast to WNT-driven tumorigenesis in colorectal cancer.

Recently, we were able to show that contextual WNT signals do influence cancer stemness-associated traits in susceptible PDAC cells [6]. To investigate WNT activity on the single cell level, we used a model system of lentivirally transduced PDAC cells with WNT-reporter constructs and isolated WNT^negative^ and WNT^positive^ populations. The latter showed more of a mesenchymal phenotype, suggesting features of epithelial to mesenchymal transition (EMT). Moreover, WNT^positive^ cells were more tumorigenic, metastatic, and chemo resistant compared to their WNT^negative^ counterparts. R-Spondin 2 (RSPO2), a critical and novel enhancer of preexisting WNT signals that binds to receptors of the leucine-rich repeat-containing G-protein coupled receptor (LGR) family, enhanced cancer stemness in susceptible PDAC cells. RSPO2 gene expression was found to be overexpressed in WNT^positive^ cells, human pancreatic spheres, gemcitabine-surviving cells, and primary murine CSCs of p48^Cre^;LSL-Kras^G12D^;p53^flox/+^ mice. WNT^positive^ CSCs in orthotopic PDAC tumor sections were surrounded by RSPO2 (unpublished data), and RSPO2 expression significantly correlated with EMT inducer ZEB1 in tissue microarrays of PDAC patients. In an attempt to simulate these contextual cues of the tumor microenvironment (TME), extrinsic addition of RSPO2 significantly increased the WNT^positive^ CSC pool, drove EMT, and activated ERK1/2 signaling (Figure 1C). Last, stimulation with RSPO2 and WNT3a led to detachment of susceptible PDAC CSCs and initiated sphere formation (unpublished data). Taken together, this artificially created environment seemed to recapitulate properties of CSC niches, moving up susceptible tumor cells on the hierarchical CSC ladder, and suggested high tumor cell plasticity (Figure 1C).

However, not all PDAC cell lines tested were amenable to extrinsic RSPO stimulation and our data corroborated the notion that PDAC is a heterogeneous disease on the intra- and intertumoral level. Along the lines, cancer stemness seemed plastic in some, but not in all PDAC with regards to canonical WNT. Nevertheless, we believe that functional testing in general and especially for RSPO/WNT-signaling to identify susceptible CSCs is necessary to extent the arsenal of therapeutic options in this fatal disease. Functional testing may be carried out by lentiviral techniques, similar to ours, the use of biomarkers such as RSPO receptors, or RSPO-enriched organoid cultures of primary PDAC tissues with subsequent drug screens.

Further research must show, whether addressing the tumor microenvironment of PDAC, e.g. by Vitamin D analogues, may also modify CSC states. Off-label use of FDA-approved substances might further amplify the armory for physicians and patients battling PDAC, and we have recently shown that WNT signaling and cancer stemness can be significantly targeted by these means [7]. Additionally, early detection of PDAC will be key to successful treatment of the disease. Diagnostics based on exosome detection and their analysis are intriguing novel instruments [8]. Functional CSC assays including pathway readouts in combination with exosome studies are, hence, exciting and innovative approaches for diagnostics and therapeutics of the future.
